# Haphephobia: a rare specific phobia of being touched

**DOI:** 10.1192/j.eurpsy.2022.989

**Published:** 2022-09-01

**Authors:** S. Nahar, M. Inam, M. Miah

**Affiliations:** 1 National Institute of Mental Health, Dhaka, Bangladesh., Adult Psychiatry, Dhaka South, Bangladesh; 2 Sylhet MAG Osmani Medical College, Psychiatry, Sylhet, Bangladesh; 3 National Institute of Mental Health, Dhaka, Bangladesh., Psychiatry, Dhaka, Bangladesh; 4 Shaheed Tajuddin Ahmed Medical College, Psychiatry, Dhaka, Bangladesh

**Keywords:** Specific phobia, Haphephobia, Anxiety disorders

## Abstract

**Introduction:**

Haphephobia is a morbid fear of being touched or touching. The symptoms of Haphephobia are very similar to other specific phobias.

**Objectives:**

Presentation of a case of haphephobia due to childhood sexual abuse

**Methods:**

Mrs. X., A 22-year-old Bangladeshi female, presents to the psychiatric consult service with an intense fear of being touched by her husband. She told that whenever her husband comes closer to her, her heart starts to pound fast, she feels discomfort in the chest, a burning sensation on her whole body, and loses control over the environment. Furthermore, she can’t sleep properly for the fear of being touched. Her in-laws’ parents concluded that some ‘evil spirits’ might cause the symptoms. So her husband brought her to a Psychiatrist. On an in-depth assessment session, ensuring all the confidentiality issues, she told the Psychiatrist that she has a history of brutal sexual abuse followed by the threat to kill her by her stepfather at the age of fourteen.

**Results:**

After a thorough medical workup and history gathering, her consultant psychiatrist could elucidate the source of the presenting picture and told her that she developed haphephobia, and suggested taking psychotherapy along with prescribed medicines.

**Conclusions:**

Fear of being touched is a particularly difficult fear to cope with. Patients with haphephobia after sexual assault should be handled very cautiously by the experts keeping confidentiality issues in mind. Cognitive-behavior therapy, Exposure therapy, Virtual reality exposure therapy, practicing mindfulness, using daily coping strategies, and medications like beta-blockers, anxiolytics, antidepressants can help a person to overcome haphephobia.

**Disclosure:**

No significant relationships.

